# A comparison of four quasi-experimental methods: an analysis of the introduction of activity-based funding in Ireland

**DOI:** 10.1186/s12913-022-08657-0

**Published:** 2022-11-03

**Authors:** Gintare Valentelyte, Conor Keegan, Jan Sorensen

**Affiliations:** 1 Structured Population and Health services Research Education (SPHeRE) Programme, School of Population Health, RCSI University of Medicine and Health Sciences, Mercer Street Lower, Dublin, Ireland; 2 Economic and Social Research Institute (ESRI), Whitaker Square, Dublin, Ireland; 3 Healthcare Outcome Research Centre (HORC), School of Population Health, RCSI University of Medicine and Health Sciences, Dublin, Ireland

**Keywords:** Interrupted time-series, Difference-in-differences, Propensity score matching, Synthetic control, Activity-based funding, Policy evaluation

## Abstract

**Background:**

Health services research often relies on quasi-experimental study designs in the estimation of treatment effects of a policy change or an intervention. The aim of this study is to compare some of the commonly used non-experimental methods in estimating intervention effects, and to highlight their relative strengths and weaknesses. We estimate the effects of Activity-Based Funding, a hospital financing reform of Irish public hospitals, introduced in 2016.

**Methods:**

We estimate and compare four analytical methods: Interrupted time series analysis, Difference-in-Differences, Propensity Score Matching Difference-in-Differences and the Synthetic Control method. Specifically, we focus on the comparison between the control-treatment methods and the non-control-treatment approach, interrupted time series analysis. Our empirical example evaluated the length of stay impact post hip replacement surgery, following the introduction of Activity-Based Funding in Ireland. We also contribute to the very limited research reporting the impacts of Activity-Based-Funding within the Irish context.

**Results:**

Interrupted time-series analysis produced statistically significant results different in interpretation, while the Difference-in-Differences, Propensity Score Matching Difference-in-Differences and Synthetic Control methods incorporating control groups, suggested no statistically significant intervention effect, on patient length of stay.

**Conclusion:**

Our analysis confirms that different analytical methods for estimating intervention effects provide different assessments of the intervention effects. It is crucial that researchers employ appropriate designs which incorporate a counterfactual framework. Such methods tend to be more robust and provide a stronger basis for evidence-based policy-making.

**Supplementary Information:**

The online version contains supplementary material available at 10.1186/s12913-022-08657-0.

## Introduction

In health services research, quasi-experimental methods continue to be the main approaches used in the identification of impacts of policy interventions. These methods provide alternatives to randomised experiments e.g. Randomised Controlled Trials (RCTs), which are less prevalent in health policy research, particularly for larger scale interventions. Examples of previously conducted experiments include the RAND Health Insurance Experiment [1] and the Oregon Health Insurance Experiment [2] which have since led to the restructuring of health insurance plan policies across the United States. Although such large-scale experiments can generate robust evidence for informing health policy decisions, they are often too complex, expensive, unethical or infeasible to implement for larger scale policies and interventions [3, 4]. Quasi-experimental methods provide an alternative means to policy evaluation, using non-experimental data sources, where randomisation is infeasible or unethical when the intervention already occurred and its evaluation occurred later [3].

The evaluation of policy impacts, regardless of analytical approach, is aimed at identifying causal effects of a policy change. A concise guide highlights the approaches which are appropriate for evaluating the impact of health policies [3]. A recent review identified a number of methods appropriate for estimating intervention effects [5]. Additionally, several control-treatment approaches have recently been compared in terms of their relative performance [6, 7].

However, there is limited empirical evidence in the health services research field comparing control-treatment analytical approaches to non-control-treatment approaches, used for estimating health intervention or policy effects. We use an empirical example of Activity-Based Funding (ABF), a hospital financing intervention, to estimate the policy impact using four non-experimental methods: Interrupted Time-Series (ITS), Difference-in-Differences (DiD), Propensity Score Matching Difference-in-Differences (PSM DiD), and Synthetic Control (SC). A review of the application of these methods in the literature examining ABF impacts has recently been undertaken [5]. Out of 19 identified studies, six studies employed ITS, seven employed DiD and one study employed the SC approach [5]. The identified effects, as assessed by reporting on a set of hospital outcomes, varied based on the analytical method that was used. The studies which employed ITS all reported statistically significant effects post-ABF which have led to increased levels of hospital activity [8, 9], and reductions in patient length of stay (LOS) [10–13]. In contrast, the evidence is more mixed, among the remaining studies which employed control-treatment methods. For example, significant increases in hospital activity were reported in three studies which used the DiD approach [14–16], while another study found no significant impacts in terms of activity [17]. Similarly, contrasting evidence in terms of changes in LOS [16, 18, 19] and mortality [18, 20] were also reported. Therefore, the overall evidence on the impacts of ABF on hospital outcomes can be considered mixed, and as highlighted by Palmer et al. (2014) [21] *‘Inferences regarding the impact of ABF are limited both by inevitable study design constraints (randomized trials of ABF are unlikely to be feasible) and by avoidable weaknesses in methodology of many studies’* [21].

The aim of this study is to compare these analytical methods in their estimation of intervention effects, using an empirical case of ABF introduction in Ireland. Specifically, we focus on the comparison of control-treatment analytical approaches (DiD, PSM DiD, SC), to ITS, a commonly used non-control-treatment approach for evaluating policies and interventions. Additionally, we contribute to the very limited research evidence assessing the impacts of ABF within the Irish context.

### ABF and the Irish health system

Activity-based funding (ABF) is a financing model that incentivises hospitals to deliver care more efficiently [22]. Under ABF, hospitals receive prospectively set payments based on the number and type of patients treated [22]. Services provided to patients are reflected by an efficient price of providing those services and adjustments incorporated for different patient populations served. Prices are determined prospectively e.g. in terms of Diagnosis Related Groups (DRGs), and reflect differences in hospital activity, based on types of diagnosis and procedures provided to patients [23]. DRGs provide transparent price differences, directly linking hospital services provision to hospital payments. In theory, this incentivises hospitals to deliver more efficient healthcare (e.g. shorten LOS) and to be more transparent in their allocation of resources and finances [22, 24].

The Irish healthcare system is predominantly a public health system, with the majority of health expenditure raised through general taxation (72%), and remainder through out-of-pocket payments (13%) and voluntary private health insurance (15%) [25]. In Ireland, most hospital care is delivered in public hospitals and this care is mostly government-financed, with approximately one-fifth of care delivered in public hospitals privately financed [25, 26]. Patients who receive private day or inpatient treatment in public hospitals are required to pay private accommodation and consultant charges. The majority of private patient activity in public hospitals is funded through private health insurance with the remainder through out-of-pocket payments. Public or private patient status relates to whether the hospital patient saw their consultant on a public or private basis [27]. For non-consultant hospital staff, the same publicly funded staff are employed in delivering care to both publicly and privately financed patients [27].

Traditionally, all Irish public hospitals were funded on a budgetary block grant basis based on historical performance, making it difficult to measure and monitor activity and funding of public hospital care [28]. On the 1st January 2016, a major financing reform was introduced, and funding of public patients in most public hospitals moved to ABF [29]. ABF was introduced centrally by the Health Services Executive (HSE), responsible for delivery of public health services in Ireland. All public inpatient activity is funded under ABF, while all outpatient and Emergency Department (ED) activity continues to be funded using block budgets [30]. The ABF funding model is based on prospectively set average DRG prices, and additionally financially penalises hospitals for long patient LOS [30]. Additionally, the amount of activity that a hospital can carry out as well as the maximum funding it can receive, is capped, to preserve the overall health budget provided to a particular hospital [30]. Public patient reimbursement is based on the average price of DRGs, in contrast to private patients who are reimbursed at a per-diem basis [30].

Thus, this key difference in reimbursement between public and private patients treated in the same hospitals, lends itself to a naturally occurring control group for our analysis using the control-treatment approaches.

## Methods

### Estimation models

#### Interrupted time-series analysis

Interrupted Time Series (ITS) analysis identifies intervention effects by comparing the level and trend of outcomes pre and post intervention [31]. Often, ITS compares outcome changes for a single population and does not specify a control group against which intervention effects can be compared [32]. This can bias the estimated intervention effects, as a defined control group often eliminates any unmeasured group or time-invariant confounders from the intervention itself [33]. Therefore, ITS can overestimate the effects of an intervention producing misleading estimation results [4].

The ITS analysis model can be presented as [34, 35],

$$\eqalign{Y}_{t }= {\beta }_{0}+ {\beta }_{1}T+ {\beta }_{2}{X}_{t}+ {\beta }_{3}T{X}_{t}+ {\epsilon }_{t}$$

Where ${Y}_{t }$is the outcome measured at time *t*, $T$ is the time since the start of the study, ${X}_{t}$ is a dummy variable representing the intervention (0 = pre-intervention period, 1 = post-intervention period), and *TX* is an interaction term; ${\beta }_{0}$ represents the intercept of the outcome (baseline level at T = 0), ${\beta }_{1}$ is the change in outcome until the introduction of the intervention (pre-intervention trend), ${\beta }_{2}$ is the change in the outcome following the intervention (the level change), ${\beta }_{3}$ represents the difference between pre-intervention and post-intervention slopes of the outcome (treatment effect over time).

#### Potential outcomes framework

Alternatively, analytical approaches such as Difference-in-Differences (DiD), Propensity Score Matching Difference-in-Differences (PSM DiD) and Synthetic Control (SC) overcome some of the shortcomings of ITS. These approaches are based on the counterfactual framework and the idea of potential outcomes which quantify the estimation of causal effects of a policy or an intervention^1^. The potential outcomes framework defines a causal effect for an individual as the difference in outcomes that would have been observed for that individual with and without being exposed to an intervention [36, 37]. Since we can never observe both potential outcomes for any one individual (we cannot go back in time to expose them to the intervention), we cannot compute the individual treatment effect [36]. Researchers therefore focus on average causal effects across populations guided by this potential outcomes framework [3, 36, 37]. Therefore in practice, estimation is always related to the counterfactual outcome, which is represented by the control group [36, 38]^2^. Consequently, it is for this reason all of these analytical approaches use a clearly defined control group in estimation, against which the outcomes for a group affected by the intervention are compared. The inclusion of a control group improves the robustness of the estimated intervention effects, by approximating experimental designs such as a RCT, the gold standard [38].

#### Difference-in-differences analysis

The DiD approach estimates causal effects by comparing the observed outcome changes pre intervention with the counterfactual outcomes post intervention, between a naturally occurring control group and a treatment group exposed to the intervention change [33]. The key advantage of the DiD approach is its use of the intervention itself as a naturally occurring experiment, allowing to eliminate any exogenous effects from events occurring simultaneously to the intervention [33, 38].

The DiD approach estimates the average treatment effect on the treated (ATT) across individual units at a particular time point, represented by the general DiD model as [3, 6, 33, 38],

$$\eqalign{{Y}_{it }= {\beta }_{0}+ {\beta }_{1}{D}_{i}+ {\beta }_{2}{X}_{t}+ {\beta }_{3}\left({D}_{i}*{X}_{t}\right)+{h}_{i} + \\{{\lambda }}_{t}+ {\epsilon }_{it}}$$

Where ${Y}_{it }$is the value of the outcome observed for unit *i* at time *t*, ${D}_{i}$ is an indicator of unit *i* being in a treatment group (vs. control group), ${X}_{t}$ is a dummy variable representing the intervention period (0 = pre-intervention period, 1 = post-intervention period), and ${D}_{i}*{X}_{t}$ is the interaction term between the two; ${\beta }_{1}$ represents the estimated average difference in *Y* between the treatment and control groups, ${\beta }_{2}$ is the expected average change in *Y* from before to after the onset of the intervention, ${\beta }_{3}$ is the DiD estimator which captures the difference in outcomes before and after the intervention between the treatment and control groups i.e. the estimated average treatment effect on the treated (ATT), ${h}_{i}$is a vector of hospital fixed effects 3 which capture unobserved time-invariant differences amongst hospitals (e.g. management), ${{\lambda }}_{t}$captures time fixed effects for each quarter *t*, and ${\epsilon }_{it}$ represents exogenous, unobserved idiosyncratic shocks.

However, DiD relies on the parallel trends assumption which states that, in the absence of treatment, the average outcomes for the treated and control groups would have followed parallel trends over time [33]. This parallel trends assumption can be represented as [33, 38],

$$\eqalign{E\left[{Y}^{0}\right(1)- {Y}^{0}(0\left) \right| D=1]=\\E[{Y}^{0}\left(1\right)- {Y}^{0}\left(0\right) | D=0]}$$

Where ${Y}^{0}\left(0\right)$ is the outcome pre-intervention observed for all units in both the treatment (D = 1) and control (D = 0) groups; ${Y}^{0}\left(1\right)$ is the outcome post-intervention observed only for the control group and represents the unobserved counterfactual for units in the treatment group (D = 1). This assumption cannot be statistically tested, as it applies to the unobserved counterfactual post-intervention [33, 38]. However, it is possible to examine the pre-treatment trends between both groups, by re-running the DiD model which includes an interaction between time and the treatment dummy, in the pre-intervention period [39].

#### Propensity score matching difference-in-differences

PSM DiD is an extension to the standard DiD approach. Using this approach, outcomes between treatment and control groups are compared, after matching them with similar observable factors, followed by estimation by DiD [40–42]. Combining the PSM approach with DiD allows further elimination of any time-invariant differences between the treatment and control groups, and allows selection on observables and unobservables which are constant over time [40, 43]. Additionally, matching on the propensity score accounts for imbalances in the distribution of the covariates between the treatment and control groups [40] 4. We present this model as follows [40],

$$\eqalign{Y= E\left({Y}_{1i|D=1}- {Y}_{1i|D=0}\right)|P\left({x}_{0i}\right)-\\E\left({Y}_{0i|D=1}- {Y}_{0i|D=0}\right)|P\left({x}_{0i}\right)}$$

Where ${Y}_{1i}$ and ${Y}_{0i}$is the outcome in the post-intervention and pre-intervention period for individual patient episode *i* respectively, ${D}_{i}=1$ indicates individual patient episode *i* is in the treatment group, ${D}_{i}=0$ indicates individual patient episode *i* is in the control group, $P\left({x}_{0i}\right)$ represents the probability of treatment assignment conditional on observed characteristics in the pre-intervention period.

In our final PSM DiD estimation model we estimate the average treatment effect on the treated (ATT) using nearest neighbour matching propensity scores, by selecting the one comparison unit i.e. patient episode whose propensity score is nearest to the treated unit in question. We present our estimation model as follows:

$$\eqalign{{Y}^{\left(PSM DiD\right)} =&\frac{1}{{N}_{{D}_{1}}} \\ & \sum _{i\in {D}_{1}\cap S}\left[\left(\genfrac{}{}{0pt}{}{ }{ }{Y}_{i,t+1}^{1}- {Y}_{i,t}^{0}\right) - \sum _{j\in {D}_{0}\cap S}{w}_{ij}\left(\genfrac{}{}{0pt}{}{ }{ }{Y}_{j,t+1}^{0}- {Y}_{j,t}^{0}\right) \right]}$$

Where ${D}_{1}$ and ${D}_{0}$ represent the treatment and control groups respectively, ${w}_{ij}$ the nearest neighbour matching weights, and *S* is the area of common covariate support^5^.

Additionally, PSM makes the parallel trends assumption more plausible as the control groups are based on similar propensity scores in the PSM DiD approach. PSM forms statistical twin pairs before conducting DiD estimation, thus increasing the credibility of the identification of the treatment effect [40]. Instead, PSM relies on the conditional independence assumption (CIA). This assumption states that, in absence of the intervention, the expected outcomes for the treated and control groups would have been the same, conditional on their past outcomes and observed characteristics pre-intervention [40, 44]. However, it is also important to note, that even if covariate balance is achieved in PSM DiD, this does not necessarily mean that there will be balance across variables that were not used to build the propensity score [40, 44]. It is for this reason that the CIA assumption is still required.

Furthermore, recent developments of the DiD approach have highlighted that additional assumptions are necessary to ensure the estimated treatment effects are unbiased [45]. It is proposed that estimates will remain consistent after conditioning on a vector of pre-treatment covariates [45]. This was our motivation for employing the PSM DiD approach, as it accounts for pre-intervention characteristics, which allow to further minimise estimation bias. PSM DiD achieves this by properly applied propensity scores, based on matched pre-intervention characteristics, thus eliminating observations that are not similar between treatment and control groups [41]. Further developments have been made to account for multiple treatment groups, which receive treatment at various time periods i.e. differential timing DiD [46]. However, this does not affect our analysis, as the introduction of ABF in our empirical example took place at one time.

#### Synthetic control

The Synthetic Control (SC) method estimates the ATT by constructing a counterfactual treatment-free outcome for the treated unit using the weighted average of available control units pre-intervention [44, 47, 48]. The weights are chosen so that the outcomes and covariates for the treated unit and the synthetic control are similar in the pre-treatment period [44, 48]. This assumption may not hold in reality, particularly when estimating policy impacts, thus alternative analytical approaches which avoid the parallel trends assumption have been considered.

The SC approach becomes particularly useful in cases when a naturally occurring control group cannot be established, or in cases where the parallel trends assumption does not hold, and can often complement other analytical approaches [48]. Similarly to PSM, the SC method also relies on the CIA, and controls for pre-treatment outcomes and covariates by re-weighting treated observations, using a semiparametric approach [44]. For a single treated unit the synthetic control is formed by finding the vector of weights *W* that minimises [44]:

$$\eqalign{ ({X}_{1}-{X}_{0}W){\prime }V({X}_{1}-{X}_{0}W)}$$

Where *W* represents the vector of weights that are positive and sum to 1, ${X}_{1}$ contains the pre-treatment outcomes and covariates for the treated unit, ${X}_{0}$ contains the pre-treatment outcomes and covariates for the control unit, and *V* is a positive matrix capturing the relative importance of the chosen variables as predictors of the outcome.

The choice of *V* is important as W* depends on the choice of *V*. The synthetic control W*(V) is meant to reproduce the behaviour of the outcome variable for the treated unit in the absence of the treatment. Often a *V* that minimises the mean squared prediction error is chosen [44, 48]:

$$\eqalign {\sum _{t=1}^{T_0}}{\left(\genfrac{}{}{0pt}{}{ }{{Y}_{1t}} - \sum _{j=2}^{J+1}{W}_{j}^{*}\left(V\right){Y}_{jt}\right)}^{2}$$

Where ${T}_{0}$ is the pre-intervention period, ${Y}_{1t}$ is the outcome post-intervention at time *t*, ${Y}_{jt}$ is the value of the covariates for unit *j* at time *t*, ${W}_{j}^{*}\left(V\right)$ is the synthetic control for unit *j*, W* is a vector of optimally chosen weights.

Similarly, we limit biases in our estimated treatment effects [45] using the SC approach, which restricts the synthetic control weights to be positive and sum to one and such that the chosen weights minimise the mean squared prediction error with respect to the outcome [49].

#### Data and methods

In our empirical example analysis, we used national Hospital In-Patient Enquiry (HIPE) administrative activity data from 2013 to 2019 for 19 public acute hospitals providing orthopaedic services in Ireland. HIPE data used in our analysis record and classify all activity (public and private) in Irish public hospitals [27]. We divided our data into quarterly time periods (n = 27) based on admission date. Data were available for 12 quarters pre-ABF introduction, and 15 quarters post-ABF introduction. We assessed the impact of ABF on patient average LOS, following elective hip replacement surgery, for a total of 19,565 hospital patient episodes.

For each analysis, we included hospital fixed effects and controlled for the same covariates: Age categories (reference category 60–69 years), average number of diagnoses, average number of additional procedures (additional to hip replacement), Diagnosis-Related Group (DRG) complexity (split by minor and major complexity) and interaction variables: Age categories by average number of diagnoses, age categories by average number of additional procedures, age categories by DRG complexity.

We estimated the ITS model using ordinary least squares and included public patient episodes only. Following guidance from previous studies [32, 50], we accounted for seasonality by including indicator variables for elapsed time since ABF introduction. Additionally, we checked for presence of autocorrelation by plotting the residuals and the partial autocorrelation function [32, 50].

For the remaining models, we used treatment and control groups consisting of public and private patient episodes, respectively, and estimated the average treatment effects on the treated (ATT). We used the key differences in reimbursement between public (DRG payments) and private (per-diem payments) patient episodes, to differentiate our treatment group from the control group. The identification strategy exploits the fact that per-diem funding of private patient care remained unchanged over the study period. Any change in outcome between public and private patients before and after the introduction of ABF should be due to the policy introduction.

In our DiD analysis, we controlled for common aggregate shock changes by including dummy variables for each time period (time fixed effects). We additionally examined the parallel trends assumption by interacting the time and treatment indicators in the pre-ABF period (see Supplementary Tables 4, Additional File 6).

We estimated PSM DiD in a number of steps^6^: First we estimated propensity scores to treatment based on our list of covariates, using a probit regression. Second, we matched the observations in the treatment group (public patient episodes) with observations in the control group (private patient episodes) as per estimated propensity scores with the common support condition imposed. Finally, we compared the changes in the average LOS of the treated and matched controls by DiD estimation.

The SC estimation^7^ was conducted at the hospital level. It has been reported that the SC approach used in our analysis works best with aggregate-level data [44, 48, 52]. We incorporated the nested option in our estimation, a fully nested optimization procedure that searches among all (diagonal) positive semidefinite matrices and sets of weights for the best fitting convex combination of the control units [44, 52]. The synthetic control group composition consisted of private patient episodes based on characteristics from 9 different public hospitals from the sample of 19 hospitals used in our analysis [see Supplementary Tables 1, Additional File 2].

To examine whether the estimated effects from all analyses still hold, we conducted sensitivity analysis and re-estimated each analytical model using trimmed LOS at 7 days (at the 90th percentile of the LOS distribution). As illustrated by the distribution of LOS in Supplementary Fig. 1, Additional File 1, this allowed for the exclusion of outlier LOS values. Additionally, to test the robustness of the estimated treatment effects, we tested the empirical strength of each model by inclusion and exclusion of certain covariates. We also examined the trends in the pre-ABF period across all DiD models, to check whether the trends were similar across the treatment and control groups.

## Results

Table 1 summarises the key descriptive statistics of the data analysed. Over the study period, the overall average LOS for this sample of patient episodes was 5.2 days (5.3 and 5.0 days for public and private patients, respectively). The majority (31.7%) of patients were aged 60–69 years (30.9% of public and 33.8% private patients, respectively). The average number of additional diagnoses was 2.5 for public and 2.1 for private patients (overall average of 2.4), and average additional procedures were 3.3 for public and 2.8 for private patients. The DRG complexity indicates that most patients (95.7%) had undergone minor complexity hip replacement surgery.

**Fig. 1:**
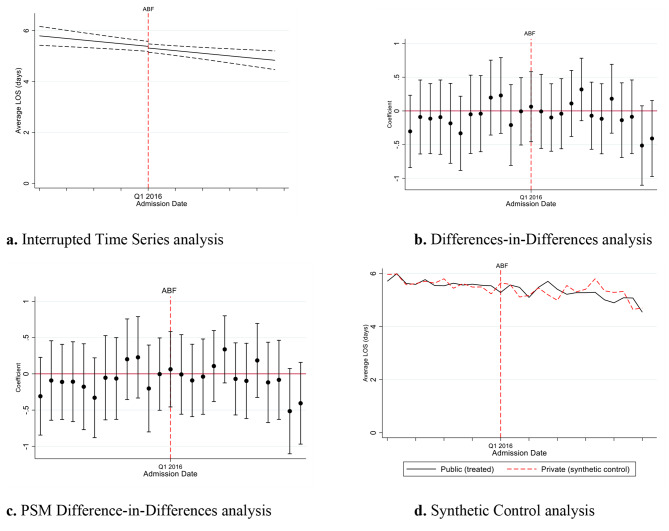
Model estimates

We illustrate the estimated intervention effects for each of the models in Fig. 1. We observe a clear reduction in the average LOS from the ITS estimates (Fig. 1a). However, the DiD and PSM DiD estimates are very similar, and we do not observe a clear effect on the average LOS, with most coefficients distributed closely around zero (Fig. 1b and c). Similarly, the SC approach could not identify a clear effect (Fig. 1d). Additionally, both the SC (Fig. 1d & Supplementary Tables 1, Additional File 2) and PSM DiD (Supplementary Fig. 2, Additional File 3) approaches achieved good balance between the treated (public patient episodes) and control (private patient episodes) groups. Our examination of the pre-ABF trends did not identify any significant differences between treatment and control groups (see Supplementary Tables 4, Additional File 6).

**Table 1:** Descriptive Statistics of key covariates used in all models by treatment and control group

	Treatment (Public)	Control (Private)	Total
**Average LOS**	5.3 days	5.0 days	5.2 days
**Age group (%)**			
**< 30** **30–39** **40–49** **50–59** **60–69** **70–79** **+ 80**	96 (0.7%) 329 (2.3%) 1026 (7.3%) 2573 (18.3%) 4338 (30.9%) 4151 (29.6%) 1530 (10.9%)	18 (0.3%) 82 (1.5%) 373 (6.8%) 982 (17.8%) 1868 (33.8%) 1633 (29.6%) 566 (10.2%)	114 (0.6%) 411 (2.1%) 1399 (7.2%) 3555 (18.2%) 6206 (31.7%) 5784 (29.6%) 2096 (10.6%)
**Average number of additional diagnoses**	2.5	2.1	2.4
**Average number of additional procedures**	3.3	2.8	3.1
**DRG complexity**			
**- major** **- minor**	13,389 (95.3%) 654 (4.7%)	5322 (96.4%) 200 (3.6%)	18,711 (95.7%) 854 (4.3%)
**N**	14,043	5522	19,565

**Fig. 2:**
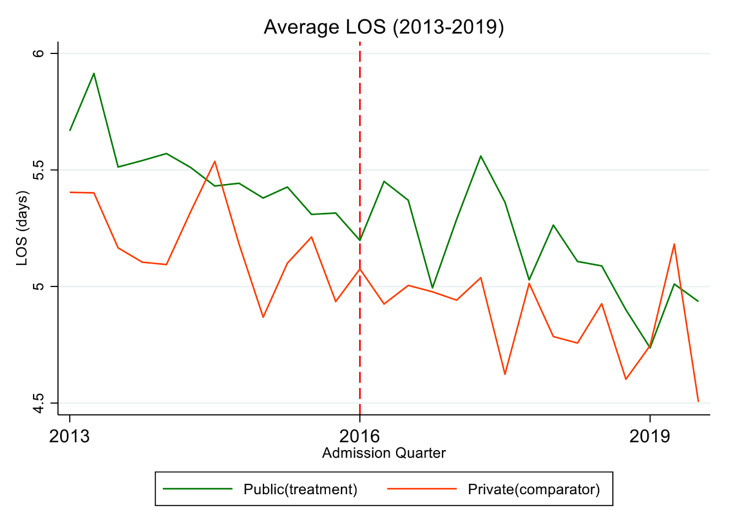
Average LOS by quarter 2013–2019 for treatment and control groups

Table 2 summarises the estimated treatment effects for each estimation model^8^. The ITS analysis suggested ABF had the largest and statistically significant impact on the average LOS for public patients, a reduction of 0.7 days (p < 0.01). However, this effect could not be observed with the control-treatment approaches, although we also see a negative but smaller effect on the average LOS from the DiD, PSM DiD and SC estimates. The effect is not statistically significant for any of these models. As illustrated in Fig. 2 below, we observe a generally declining trend in the average LOS for both the public and private patients in our data. This explains the statistically significant effects of ITS, relative to the control-treatment methods, which differentiate out the average LOS effects between both public and private patient episodes.

**Table 2:** Estimated Treatment Effects by estimation model

Estimation Model	Estimated Treatment Effect (Std. Error)	t	p-value	R^2^	Observations	FE
**ITS**	-0.734 (0.162)	-4.54	0.001***	0.43	N = 14,043	Yes
**DiD**	-0.012 (0.280)	-0.04	0.966	0.42	N = 19,565	Yes
**PSM DiD**	-0.013 (0.279)	-0.05	0.961	0.42	N = 19,549^*a*^	Yes
**SC**	-0.053 (0.136)^*b*^	-	0.400	-	N = 28^*c*^	-

Note: The treatment effect parameters for ITS, DiD and PSM DiD all indicate a marginal change (reduction) in the average LOS. The DiD and PSM DiD estimates are almost identical, indicating that the matched propensity scores did not have a substantial impact on the overall DiD estimates. In contrast, the difference between ITS and DiD estimates is substantial. All models control for hospital Fixed Effects (FE) except for SC estimation at the hospital level; ^*a*^16 observations in the treatment group were not matched; ^*b*^The SC method relies on minimising the RMSPE; ^*c*^ due to aggregated data at hospital level; Significance level: *** ; p < 0.01; robust standard errors in parenthesis

The results from our sensitivity analysis (Supplementary Tables 2, Additional File 4) revealed no material change for the ITS estimates, which remained statistically significant (p < 0.001). The estimated treatment effects from the control-treatment approaches remained small, and not statistically significant. Similarly, additional robustness testing of the estimated treatment effects by each model (and pre-ABF trend examination) remained consistent with the main results (Supplementary Tables 3, Additional File 5).

## Discussion

In this study we compared the key analytical methods that have been used in the evaluation of policy interventions and used the introduction of Activity-Based Funding (ABF) in Irish public hospitals as an illustrative policy case. Specifically, we compared several control-treatment methods (DiD, PSM DiD, SC), to a non-control-treatment approach, ITS. We contribute to the limited empirical evidence in the health services research field comparing control-treatment analytical approaches to non-control-treatment approaches, based on recent evidence highlighting the common use of these methods in estimation of health intervention or policy effects [5]. Additionally, we contribute to the very limited research evidence on the evaluation of the ABF policy within the Irish context. We were able to utilise an important dimension of the funding changes, by exploiting the fact that both publicly and privately financed patients are treated in public hospitals in Ireland and over the period of analysis, private patients were not subject to a change in their funding.

From our comparative methods analysis, ITS produced statistically significant estimates, indicating a reduction in LOS post ABF introduction, relative to control-treatment approaches, which did not indicate any significant effects. This is in line with the results from other studies, which have estimated ABF effects using ITS, and have reported significant reductions in LOS [10–13]. Caution should be taken when considering ITS, as the estimates may not truly capture the effects of the intervention of interest. This could lead to incorrect inferences, and potentially to misguided assessment of impacts from policy changes across the hospital sector. For instance, the estimated reduction in LOS for Irish public patients, may incorrectly indicate that the ABF reform has been successful. From a policy perspective, the importance of the resulting ABF effects, would be informed by the size of ITS estimates, providing potentially misleading evidence on the funding reform.

Further, caution should be taken, as ITS analysis does not include a control group, relative to the other methods we considered which incorporated a control and treatment groups. Therefore the conclusions drawn from the ITS analysis will differ to those drawn from the control-treatment approaches. Additionally, our findings from ITS analysis align with a recent study which tested the empirical strength of the ITS approach, by comparing the estimated ITS results to the results from a RCT [4]. Relative to a RCT, ITS produced misleading results, primarily driven by the lack of control group, and ITS model assumptions [4]. This would suggest, a comparison of the slope of outcomes before and after an intervention may lead to biased estimates when evaluating causal effects on outcomes affected over time, due to influences by simultaneous and other unobservable factors at the time of the intervention.

However, over the study period, the average LOS for both public (treatment) and private (control) patient cases shows a reducing trend over time (Fig. 2). By limiting the analysis to the public patients only, the ITS approach ignores the system level effect for all patients treated (public and private), across public hospitals, and picks up a statistically significant and negative effect. In contrast, the control-treatment approaches account for the simultaneous downward trend in private (control) patient activity, thus approximating a natural experiment (e.g. a RCT) more closely, and producing more robust estimates, relative to ITS.

It is important to note that often no comparison group may be available, limiting the analysis to the ITS approach. This may be driven by various data limitations. For example, the data available over a period may only partially be available for a specific intervention. Therefore, conventional regression modelling may be the only feasible approach to account for pre-intervention differences, even though there is evidence that these methods may provide biased results, most notably in the presence of time-dependent confounders [4]. Additionally, certain intervention and policy evaluations may not be feasible under a control-treatment design, and for which the ITS approach is more suitable. This applies to studies which focus on a specific patient [53] or hospital group [10], or policies at a more aggregate or population level [54], for which it is difficult to identify a naturally occurring control group. Therefore, the inclusion of a control group in these instances would not be suitable, suggesting a before-after comparison in the level and trend of outcomes using ITS analysis as a more suitable approach. Additionally, ITS models may be more effective in the evaluation of policy and intervention effects when the control-treatment specific assumptions of parallel trends and the common independence assumptions do not hold [55].

Additionally, ITS has been highlighted as an effective approach to study short-term policy and intervention effects, as estimation of long-term effects can be biased due to the presence of simultaneous shocks to the outcome of interest [56]. In contrast, control-treatment approaches such as DiD and SC have been recognised as more appropriate and robust for estimation of long-term intervention effects [57], as these allow intervention effects to change over time [38, 49]. Despite recent improvements and developments of the ITS approach [34, 35], the benefits of adopting control-treatment approaches for health intervention and policy evaluation, have been previously highlighted [33].

It should be noted that all of the methods applied in this study are limited to the evaluation of a single policy. Therefore, any other smaller scale simultaneous policies that are implemented during the period of analysis are difficult to differentiate out in many instances. However, the control-treatment methods account for any unmeasured group or time-invariant confounders from the main intervention itself by incorporating a control group [33]. For example, the introduction of ABF in our empirical example may have been accompanied by a hospital-wide discharge policy aimed at reducing LOS. In this instance, ITS may attribute the reduction in LOS as the impact of ABF entirely, although this is a hospital policy effect. Alternatively, the inclusion of a control group (e.g. patients targeted in the LOS policy, but not to ABF) would difference out the ABF effect from the LOS policy, and would capture effects specific to ABF introduction. In this case, ITS may overestimate the impacts of ABF relative to the other approaches and may further contribute to different evidence base for policy decisions.

This study has several limitations. First, we limited our ITS analysis to a single group (public patient episodes) despite recent developments to ITS for multiple group comparisons [34]. However, this was informed by a recent review, which identified that ITS was employed to estimate intervention effects for a single group [5]. Second, for each of the control-treatment methods, we assumed that any individual shocks following ABF introduction had the same expected effect on the average LOS for the treatment and control groups. Third, we assumed that all of the models were correctly specified in terms of their respective identification and functional form assumptions. However, if either the identification or the functional assumptions are violated, the estimates can be biased, particularly as highlighted in recent literature on DiD approaches [45]. Fourth, we limited our focus on two key assumptions applicable to the quasi-experimental approaches i.e. parallel trends and conditional independence, and did not focus on other assumptions e.g. common shock assumption. Fifth, recent research evidence has addressed the issues related to intervention ‘spillover effects’ i.e. the unintended consequences of health-related interventions beyond those initially intended [58]. It is possible that the differing estimated effects, based on the analytical method used, may have, or could lead to spillover effects as a result. However, given the nature of the data used in our analysis, and our focus on a single procedure in our empirical analysis, it is difficult to identify any potential spillover effects, which may have been linked to ABF. More exploration of such effects may be necessary in future research. Finally, caution should be taken in generalising the reported ABF effects in this study given that our empirical example focused on one procedural group in one particular country.

## Conclusion

In health services research it is not always feasible to conduct experimental analysis and we therefore often rely on observational analysis to identify the impact of policy interventions. We demonstrated that ITS analysis produces results different in interpretation relative to control-treatment approaches such as DiD, PSM DiD and SC. Our comparative method analysis therefore suggests that choice of analytical method should be carefully considered and researchers should strive to employ more appropriate designs incorporating control and treatment groups. These methods are more robust and provide a stronger basis for evidence-based policy-making and evidence for informing future financing reform and policy.

## Electronic supplementary material

Below is the link to the electronic supplementary material.

Supplementary Material 1

## Data Availability

The data that support the findings of this study were made available under a strict user agreement with the Healthcare Pricing Office. Access to the data may only be sought directly from the Healthcare Pricing Office.

## List of abbreviations

RCT: Randomised Controlled Trial
ITS: Interrupted Time Series
DiD: Difference-in-Differences
PSM: Propensity Score Matching
PSM DiD: Propensity Score Matching Difference-in-Differences
SC: Synthetic Control
CIA: Conditional Independence Assumption
ABF: Activity-Based Funding
ATT: Average Treatment effect on the Treated
HSE: Health Service Executive
HIPE: Hospital In-Patient Enquiry
LOS: Length of Stay
DRG: Diagnosis-Related Group

## Footnotes

1 The treatment effect in terms of potential outcomes: where Y^*0*^ (*i,t*) is the outcome that individual *i* would attain at time *t* in absence of treatment and Y^*1*^ (*i,t*) is the outcome that individual *i* would attain at time *t* if exposed to treatment. The treatment effect on the outcome for individual *i* at time *t* is: Y^*1*^(*i,t*) – Y^*0*^(*i,t*). The fundamental identification problem is that for any individual *i* and time *t*, both potential outcomes Y^*0*^(*i, t*) and Y^*1*^(*i, t*) are not observed and we cannot compute the individual treatment effect. We only observe the outcome Y(*i, t*) expressed as: Y(*i, t*) = Y^*0*^(*i, t*)(1 − D(*i, t*)) + Y^*1*^(*i, t*)D(*i, t*), [D(*i,t*) = 0 control and D(*i,t*) = 1 treatment]. Since treatment occurs after period t = 0, we can denote D(*i*) = D(*i, 1*), then we have Y(*i, 0*) = Y^*0*^(*i, 0*) and Y(*i, 1*) = Y^*0*^(*i, 1*)(1 − D(*i*)) + Y^*1*^(*i, 1*)D(i) (Rubin (1974)).

2 The change in outcomes from pre to post-intervention in the control group is a proxy for the counterfactual change in untreated potential outcomes in the treatment group.

3 The unit used is at discharge level but we only have one observation per discharge by definition therefore we cannot apply discharge fixed effects and instead have to include hospital fixed effects.

4 Matching on the propensity score works because it imposes the same distribution of the covariates for both the control and treatment groups (Rosenbaum and Rubin (1983)).

5 The common support condition guarantees that only units with suitable control cases are considered by dropping treatment observations whose propensity score is higher than the maximum or less than the minimum propensity score of the controls.

6 Using the *psmatch2* Stata command using nearest neighbour matching which showed the best balancing properties after comparing several algorithms [51].

7 Using the *synth* Stata command [44, 52].

8 Reported p-values for ITS and DiD are for the hypothesis that ATT = 0. For DiD PSM, reported p-values are conditional on the matched data. For SC, reported p-values were calculated using placebo-tests in a procedure akin to permutation tests (Abadie et al. 2010). This involved iteratively resampling from the control pool, and in each iteration re-assigning each control unit as a ‘placebo treated unit’, with a probability according to the proportion of treated units in the original sample. The synthetic control method was then applied on these ‘placebo data’ and ATT calculated for the placebo treated versus control units. The p-value for the ATT was calculated according to the proportion of the replicates in which the absolute value of the placebo-ATT exceeded the estimated ATT. It should be noted that the p-value based on placebo tests relate to falsification tests, while the p-values reported for the other methods relate to sampling uncertainty. Hence the p-values between each estimated model are not directly comparable.
