# Supplementary material for: A comparison of four quasi-experimental methods: an analysis of the introduction of activity-based funding in Ireland
Source: BMC Health Serv Res. 2022 Nov 3;22:1311. doi: 10.1186/s12913-022-08657-0 (PMC9635092; doi:10.1186/s12913-022-08657-0)
Supplement: Supplementary file 1 — Supplementary Material 1 [file 12913_2022_8657_MOESM1_ESM.docx]

**Additional Files – All combined**

**Additional File 1**

**Supplementary Figure 1.** Distribution of LOS

**Additional File 2**

**Supplementary Table 1.** Composition of the Synthetic Control

| Control Units | Unit Weight |
| --- | --- |
|  |  |
| 1 | 0.518 |
| 2 | 0.147 |
| 3 | 0.096 |
| 4 | 0.083 |
| 5 | 0.071 |
| 6 | 0.058 |
| 7 | 0.025 |
| 8 | 0.001 |
| 9 | 0.001 |

**Additional File 3**

**Supplementary Figure 2.** PSM balance

**Additional File 4**

**Supplementary Table 2.** Estimated Treatment Effects by estimation model – with trimmed LOS at 7 days

| **Estimation Model** | **Estimated Treatment Effect**  **(Std. Error)** | **t** | **p-value** | **R^2^** | **Observations** | **FE** |
| --- | --- | --- | --- | --- | --- | --- |
| **ITS** | -0.298  (0.081) | -3.68 | 0.001*** | 0.32 | N=12,066 | Yes |
| **DiD** | -0.021  (0.087) | -0.25 | 0.806 | 0.32 | N=17,067 | Yes |
| **PSM DiD** | -0.017  (0.087) | -0.19 | 0.848 | 0.32 | N=17,047*^a^* | Yes |
| **SC** | -0.048  (0.170) *^b^* | - | 0.500 | - | N=280 *^c^* | - |

Note: All models control for hospital Fixed Effects (FE) except for SC estimation at the hospital level; *^a^* 20 observations in the treatment group were not matched; *^b^* The SC method relies on minimising the RMSPE; *^c^* due to aggregated data at hospital level; Significance level: *** ; p<0.01; robust standard errors in parenthesis

**Additional File 5.**

**Supplementary Table 3.** Sensitivity analysis – robustness of treatment effects to alternative combinations of covariates

| **Estimation Model** | **ITS** | | | | **DiD** ^b^ | | | | **PSM DiD** ^a^ | | | | | **SC** | |
| --- | --- | --- | --- | --- | --- | --- | --- | --- | --- | --- | --- | --- | --- | --- | --- |
|  | (1) | (2) | (3) | (4) | (1) | (2) | (3) | (4) | (1) | (2) | (3) | (4) | (1) | | (2) |
| **Treatment Effect** | -0.371  (0.049)^***^ | -0.367  (0.049)^***^ | -0.675  (0.167)^***^ | -0.348  (0.046)^***^ | -0.065 (0.081) | -0.064  (0.080) | -0.066  (0.080) | -0.015  (0.074) | -0.065  (0.081) | -0.067  (0.080) | -0.069  (0.080) | -0.021  (0.075) | -0.044  (0.116) *^b^* | | -0.076  (0.127) |
| **Patient covariates** | Yes | Yes | Yes | Yes | Yes | Yes | Yes | Yes | Yes | Yes | Yes | Yes | Yes | | No |
| **Interaction covariates** | No | Yes | Yes | Yes | No | Yes | Yes | Yes | No | Yes | Yes | Yes | No | | Yes |
| **Time FE** | No | No | Yes | No | No | No | Yes | No | No | No | Yes | No | - | | - |
| **Hospital FE** | No | No | No | Yes | No | No | No | Yes | No | No | No | Yes | - | | - |
| **R^2^** | 0.314 | 0.324 | 0.326 | 0.423 | 0.306 | 0.316 | 0.318 | 0.414 | 0.305 | 0.316 | 0.318 | 0.413 | - | | - |
| **N** | 14,043 | 14,043 | 14,043 | 14,043 | 19,565 | 19,565 | 19,565 | 19,565 | 19,549 | 19,549 | 19,549 | 19,549 | 280 *^c^* | | 280 |

Note: PSM DiD: Estimation is using matched data with inclusion/exclusion of specified covariates; *^a^*16 observations in the treatment group were not matched; *^b^* The SC method relies on minimising the RMSPE; *^c^* due to aggregated data at hospital level; Significance level: *** ; p<0.01; robust standard errors in parenthesis. *^b^* Additional tests of parallel trends in the pre-ABF period were conducted for each DiD model: No parallel trends violations could be identified for any of the models.

**Additional File 6.**

**Supplementary Table 4.** Examination of pre-ABF trends between treatment and control groups

| **Coefficient** | **Estimated treatment effect**  **(SE)** | **t** | **p-value** | **Observations** |
| --- | --- | --- | --- | --- |
| **DiD** | -0.010  (0.260) | -0.03 | 0.89 | n= 8,982 |
| **PSM-DiD** | -0.009  (0.250) | -0.02 | 0.87 | n= 8,966 |

*Note:* estimated by interacting the time and treatment dummy in the pre-ABF period. A non-statistically significant DiD coefficient suggests no differences in the pre-treatment outcome trends between the treatment and control groups.
